# Incident diabetes within the first two years after SARS-CoV-2 infection: a population-based retrospective cohort study of the Agency for Health Protection of Milan, Italy

**DOI:** 10.1186/s12879-026-13467-4

**Published:** 2026-05-11

**Authors:** Cristina Mazzali, Pietro Magnoni, Maria Grazia Valsecchi, Daria Vigani, Claudio Lucifora, Antonio Giampiero Russo

**Affiliations:** 1 Epidemiology Unit, Agency for Health Protection of Milan, Via Conca del Naviglio 45, 20123 Milan, MI Italy; 2 School of Medicine and Surgery and Bicocca Bioinformatics Biostatistics and Bioimaging Centre (B4), University of Milano-Bicocca, Milan, Italy; 3 Department of Economics and Finance, Catholic University of the Sacred Heart, Milan, Italy; 4 Department of Economics and Management, University of Pavia, Pavia, Italy; 5 Epidemiology Unit, Agency for Health Protection Pavia, Pavia, Italy; 6 Epidemiology Unit, Agency for Health Protection Val Padana, Cremona, Italy; 7 Epidemiology Unit, Agency for Health Protection Bergamo, Bergamo, Italy; 8 Epidemiology Unit, Agency for Health Protection Brianza, Monza, Italy; 9 Epidemiology Unit, Agency for Health Protection Brescia, Brescia, Italy; 10 Epidemiology Unit, Agency for Health Protection Montagna, Sondrio, Italy; 11 Epidemiology Unit, Agency for Health Protection Insubria, Varese, Italy; 12 Respiratory Rehabilitation Unit, ASST Crema – Ospedale Santa Marta Rivolta D’Adda, Crema, Italy; 13 General Medicine Unit, ASST del Garda – Presidio Ospedaliero di Manerbio/Leno, Brescia, Italy; 14 Neurology Unit, ASST Lodi – Ospedale Maggiore di Lodi, Lodi, Italy; 15 Infectious Diseases Unit, ASST Ovest Milanese – Ospedale di Legnano, Legnano, Italy; 16 Internal Medicine Unit, ASST Pavia – Ospedale Civile di Voghera, Voghera, Italy; 17 ASST Valcamonica, Brescia, Italy; 18 Fondazione IRCCS Policlinico San Matteo, Pavia, Italy

**Keywords:** SARS-CoV-2, Diabetes, PASC, Long Covid, Epidemiology, Population cohort study

## Abstract

**Background:**

Postacute sequelae of SARS-CoV-2 infection (PASC), including persistent symptoms and acute and chronic diagnoses, have become a major research focus. Diabetes mellitus, beyond its established link to COVID-19 severity, is increasingly recognized as a potential long-term outcome. This study investigated the association between SARS-CoV-2 infection and incident diabetes using population-level health administrative data (HAD) from the Agency for Health Protection of Milan, where the epicenter of the pandemic in Italy took place.

**Methods:**

This retrospective cohort study included adult residents without a history of diabetes who underwent SARS-CoV-2 testing between 1 March and 31 December 2020. Test-positive individuals were matched 1:1 to test-negative individuals based on sex, age, and testing week. The cohort was followed through 31 December 2021. The incidence of diabetes, identified using an HAD-based case-detection algorithm, was compared between the two groups, and in stratified analyses by sex and age, using weighted Cox models adjusted for chronic comorbidities, area-level deprivation, influenza and pneumococcal vaccinations. Weights were calculated via the inverse probability weighting approach. Effect estimates are presented as hazard ratios (HRs).

**Results:**

Our final cohort included 248,176 residents (124,026 test-negative, 124,150 test-positive). Over a median follow-up time of 415 days, 739 positive (0.60%) and 657 negative (0.53%) individuals were newly identified with diabetes. The incidence among positive individuals was 572.82 per 100,000 person-years (CI 531.52–614.12), and that among negative individuals was 509.50 per 100,000 person-years (CI 470.54–548.46). The overall HR was 1.13 (CI 1.02–1.25). In stratified analyses, this effect was prominent in women aged 41–60 years (HR 1.31; CI 1.02–1.68).

**Conclusions:**

This study provides population-based evidence supporting an association between SARS-CoV-2 infection and newly detected diabetes. These findings contribute to understanding the long-term health impact of COVID-19 and may inform public health strategies for PASC prevention and management.

**Supplementary Information:**

The online version contains supplementary material available at 10.1186/s12879-026-13467-4.

## Introduction

Over the past few years, a substantial body of research has focused on the long-term consequences of SARS-CoV-2 infection and its resulting disease. Effects that extend beyond the normal postviral recovery period include specific persistent symptoms and clinical sequelae affecting several organ systems [1–3]. This phenomenon is referred to by a variety of terms, most notably, long COVID, postacute sequelae of SARS-CoV-2 infection (PASC), and post-COVID-19 condition (PCC) [4]. Several studies have analyzed a wide range of conditions potentially related to the infection at the population level [1–3, 5–8]. The associations between diabetes mellitus and mortality in individuals affected by COVID-19 are well known [9], as are poor disease management and adherence to follow-up [10] and reduced quality of life in survivors [11]. However, diabetes is also emerging as one of the long-term conditions associated with infection. To the best of our knowledge, only a few studies have specifically investigated diabetes at the population level [12, 13].

In the analysis of long-term sequelae of infection, many studies note limited generalizability of findings due to nonstandardized designs, small sample sizes, and a lack of adequate control groups [2, 5, 6]. The studied populations vary significantly, from individuals who were not hospitalized for COVID-19 [1, 6] to infected individuals with a low risk of developing severe forms of disease [2, 5, 13], up to the general population [12]. While exposure is defined by a positive test result, control groups are not always specifically defined as individuals who tested negative and were never previously positive. This is a necessary condition to differentiate the impact of SARS-CoV-2 infection from that of the disease under study [7]. With respect to the follow-up period, Naveed et al. [12] and Xie et al. [13] specifically examined long-term diabetes, with median follow-up times after infection ranging between 250 and 350 days. Mizrahi et al. [5] reported that SARS-CoV-2 infection was significantly associated with diabetes in the period 180–360 days after infection, whereas the same was not observed during the 90–180-day period. As Korompoki et al. noted [14], long-term follow-up is needed to evaluate the late onset of diabetes.

The study of diseases in populations requires the use of linkable data, which are routinely collected over extended periods, enabling the rapid identification of morbidities, life status, sociodemographic characteristics, and exposures within an entire resident population. Lombardy, in northern Italy, was one of the regions most affected by the COVID-19 pandemic, both in terms of deaths and strain on the healthcare system. The first confirmed case of person-to-person transmission of COVID-19 in Italy was diagnosed on 20 February 2020 in Codogno (Lodi Province, Lombardy) [15]. On 31 March 2022, the state of emergency in Italy was declared over. In the following months, the requirement for the green pass expired, which certified vaccination status or a negative test result for access to most public places. The high burden of COVID-19 in Lombardy during this period provides an optimal setting to study the long-term consequences of the infection using detailed population-level records that track health outcomes over time. This study aimed to evaluate the association between SARS-CoV-2 infection and long-term diabetes in a large adult population through a cohort design with a rigorous research and analysis protocol to control for confounding factors.

## Methods

### Study setting and data sources

The study is based on health administrative databases (HADs) available to the Epidemiology Unit of the ATS of Milan. Within the context of the Lombard health system, the Agencies for Health Protection (*Agenzie di Tutela della Salute*, ATS) have functions of health planning, healthcare service control, and epidemiologic surveillance. The ATS has statutory access to routinely collected health administrative data (HAD) on hospital admissions, outpatient services, exemptions from copayment, and drug prescriptions, which can be used to determine residents’ disease status. The ATS also has access to demographic databases that include information on age, sex, vital status as well as the periods during which individuals resided within the ATS territory, allowing their health outcomes to be tracked in long-term longitudinal studies. Since the first positive individuals were reported, the ATS of Milan activated a web-based system to systematically collect and store the dates and results of antigenic and molecular SARS-CoV-2 tests, submitted daily by testing laboratories (Supplementary Figure S1). The completeness of the database is further ensured by the use of data for the quarantine obligations of positive individuals. These conditions enable the ATS to follow the infections of all residents since the earliest stages of the pandemic, unless there are (likely asymptomatic) individuals who did not require a green pass and did not undergo testing.

The population under study is that residing in the territory of the ATS of Milan. The ATS of Milan covers 193 municipalities in the provinces of Milan and Lodi, with a resident population of approximately 3.5 million individuals. The catchment area comprises both smaller towns and semi-rural areas in the provinces of Milan and Lodi, as well as the metropolitan city of Milan, a highly urbanized setting with standardized access to regional healthcare services. Residents in the ATS of Milan account for about 35% of the population of Lombardy, the most populous region in Italy with approximately 10 million inhabitants. While the mean age in the ATS is comparable to that of Lombardy and Italy (about 46.1 years vs. 46.4 and 46.9, respectively), the proportion of residents aged 65 or older is slightly lower (about 23.2% vs. 23.8% and 24.7%, respectively).

### Study design and population

This was a retrospective cohort study including individuals born before 2003 who began residing in the territory of the ATS of Milan before 1 March 2019 and were still resident until death or 1 January 2021. Our study design was informed by that used by Mizrahi et al. [5].

We considered tests for the detection of SARS-CoV-2 that were carried out between 1 March 2020 and 31 December 2020 on the residents. The choice of the enrollment window was based on the peak circulation of the alpha variant and motivated by the aim of analyzing an immunologically naïve population prior to the introduction of vaccines and the circulation of different viral variants. Tests were excluded if the person had prevalent diabetes at the time of testing, if diabetes was identified within 30 days after testing, or if the person did not survive beyond 30 days after testing.

All individuals who tested positive or weakly positive were classified as exposed and enrolled in the study on the date of the first positive test. Any subsequent positive tests within 90 days from this first date are classified as part of the same clinical episode. Individuals with a negative test result, never preceded by a positive test result, were considered potential unexposed controls. Exact 1:1 matching was performed according to sex, year of birth, and week of test administration [5]. The negative test was randomly selected from the whole pool of available negative tests. Once the negative test was selected, the participant’s other negative tests were no longer considered, ensuring matching without replacement.

The end of the study was set at 31 December 2021, guaranteeing that all participants had a potential follow-up of at least one year. The start of follow-up for both positive and negative individuals was the date of the test. The Lombardy Region demographic database is updated monthly with information on changes of residence within the Region, as well as movements into and out of the Region. This allows precise tracking of residents’ periods of residence. Individuals who moved their residence outside the catchment area of ATS Milan were censored as lost to follow-up. The follow-up period for the matched pair ended when the first of the following conditions was met: diabetes detection for either of the two individuals, end of the study period, loss to follow-up of either of the two individuals, death of either of the two individuals, infection of the control defined by a positive test, and reinfection of the positive individual (defined as a new positive test more than 90 days after the first date of infection). When a control became test-positive within the enrollment period, they reentered the study as a case and were paired with a new control.

### Outcome and covariates

Diabetes and other chronic diseases considered as potential confounders (renal disease, cardiovascular diseases, respiratory diseases, and cancer) were identified using HAD-based case-detection algorithms following the specifications introduced in the Lombardy Region through Regional Council Resolution no. X/6164 of 30 January 2017, as amended and supplemented [16]. The definition of chronic conditions makes integrated use of the following currently available HADs: hospital discharge records (HDRs), which collect information about every patient discharged from public and accredited private hospitals. Diagnoses at discharge and procedures performed (if any) are coded according to the Italian version of the International Classification of Diseases − 9th revision - Clinical Modification (ICD-9-CM); database of exemptions from copayment, with reasons (chronic disease, age under 18 or over 65, low income, legal disability); database of outpatient services; drug dispensing databases, including medications distributed through territorial channels as well as hospital-dispensed drugs for outpatients. Dispensations are classified via the Anatomical Therapeutic Chemical (ATC)/Defined Daily Dose (DDD) system by the World Health Organization. The criteria for the attribution of a chronic disease consider outpatient services and drug dispensations with a lookback of one year, hospital discharge records with a lookback of five years, and active copayment exemptions with a lookback of ten years. Whenever the criteria for the detection of a disease are met, the date of the earliest fulfilling criterion is registered as the date of disease diagnosis. To identify the pathologies and more correctly attribute the date of diagnosis, the comorbidity datasets produced from 2017 to 2021 were used. Individuals are classified as diabetic if they met at least one of the following criteria: at least one hospitalization for diabetes or diabetes-related complications over the previous five years; active exemption for diabetes over the previous ten years; or antidiabetic drug therapy (either insulin or other blood glucose lowering drugs) in that year with dispensed defined daily doses covering at least half of the days from the initial dispensation to the end of the year. More technical details are presented in Supplementary Table S1.

Obesity was added as an additional clinical covariate. Its identification was based on hospitalization data by searching for the diagnosis code 278.0 in any hospital discharge record since 2013.

The most recent deprivation index, as calculated by Rosano et al. [17] for the year 2011, was employed to assess the socioeconomic status of the participants. The index is available at the level of the census section, which represents the basic territorial unit of the municipality. However, it should be noted that the geolocation of the residential address is only available for residents of the municipality of Milan but not for residents of other municipalities. Consequently, the decision was made to employ the mean value of the deprivation index of the municipality of residence outside Milan and of the census area for residents in Milan. The census areas were delineated by the Italian National Institute of Statistics through the aggregation of census sections, resulting in areas encompassing a population generally ranging from 13,000 to 18,000.

Data related to influenza vaccinations from 2017 to 2019 were considered, as were data related to pneumococcal vaccinations. COVID-19 vaccines were available starting in January 2021, after the enrollment period was over. During follow-up, the enrolled individuals could receive a maximum of two doses of the vaccine. The vaccination status was categorized according to the number of doses received as follows: fully vaccinated (two doses), partially vaccinated (single dose) and unvaccinated.

**Fig. 1:**
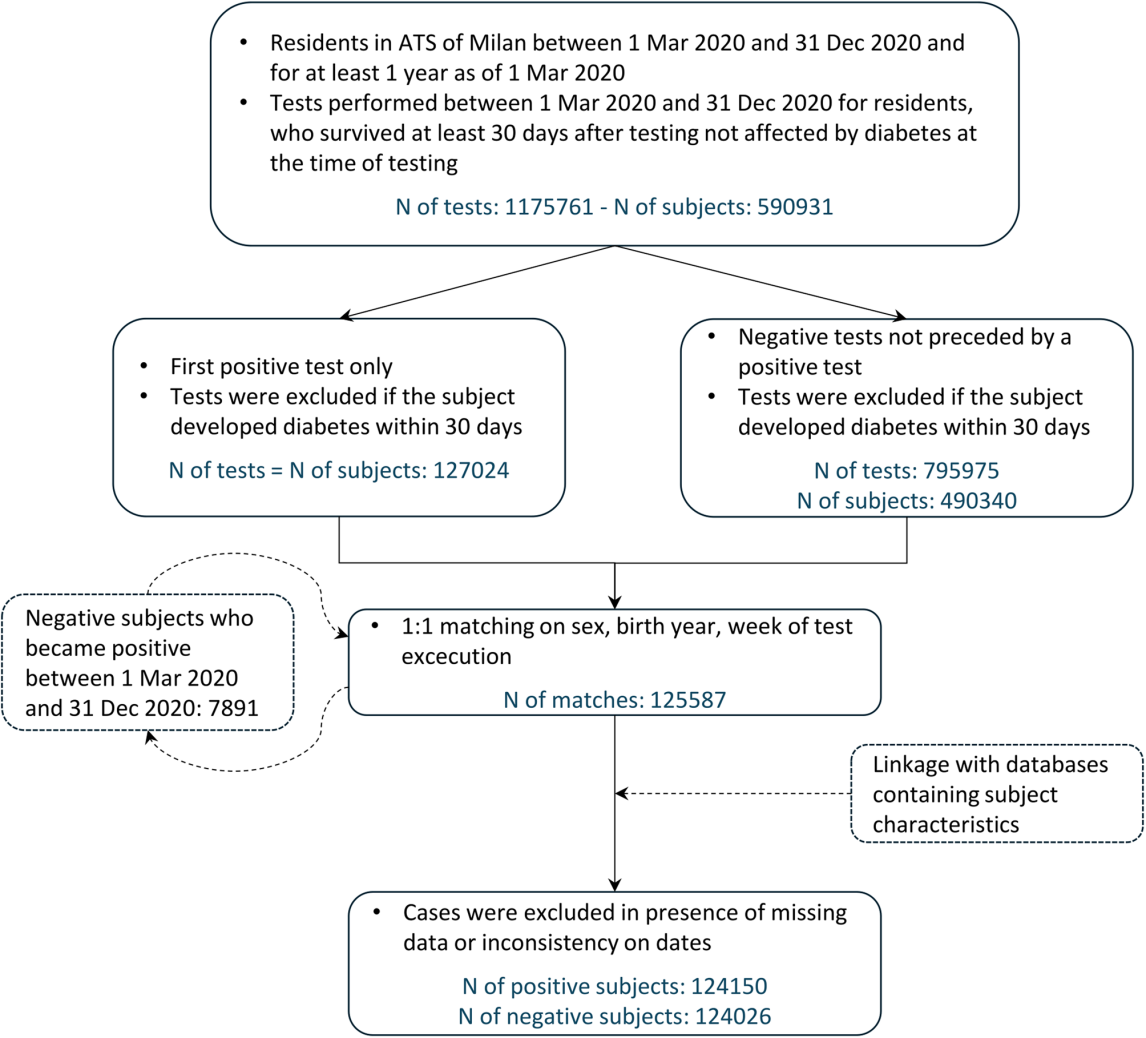
Study population and cohort selection

All the information was linked at the individual level with a deterministic linkage on the pseudonymized fiscal code. Data on diabetes diagnoses were linked with the resident cohort to exclude prevalent cases of diabetes while the study cohort was constructed (Fig. 1). All the other covariates were then linked with the individuals enrolled in the study.

### Statistical analysis

Baseline characteristics at the time of testing for positive and negative individuals were described in terms of the absolute number (percentage) for discrete variables and in terms of the mean and standard deviation (SD) or median and interquartile range (IQR) for continuous variables.

The incidence (number of events/100,000 person-years) was evaluated by considering the number of events and total time at risk at the end of the study for positive and negative participants.

The cumulative incidence was calculated through the weighted Kaplan-Meier method. The effect of exposure to SARS-CoV-2 on diabetes incidence was described with cumulative incidence curves and evaluated via a weighted Cox model [18] with exposure (positive vs. negative test) as the independent variable. Weights were calculated with the inverse probability weighting (IPW) approach via a logistic model to assess the probability of infection. We considered, as predictors in the logistic model, prespecified variables that might be associated with a positive test or diabetes onset. Specifically, we considered the comorbidities presented in Table 1, the mean deprivation index calculated at the census area level, the history of influenza vaccination over the previous three years, and the pneumococcal vaccination status. In the IPW adjustment we only considered patient characteristics associated with the outcome or affecting the relationship between exposure and outcome at the time of enrollment. COVID-19 vaccination status was used in descriptive analyses but excluded from the models, as vaccinations were introduced after the end of the enrollment period. IPW was used to generate a pseudo-population in which the distribution of measured baseline covariates was independent of exposure. A robust sandwich estimator of variance was used to account for the weighting in the Cox model. Covariate balance was assessed via mean standardized differences and variance ratios, as well as graphically.

**Table 1:** Baseline characteristics of the two study groups. The values are presented as numbers (percentages) unless stated otherwise. Standardized mean differences were calculated for confounders used in the IPW model, whereas exact 1:1 matching was performed on sex and age

Baseline characteristics	SARS-CoV-2 negative (*n* = 124026)	SARS-CoV-2 positive (*n* = 124150)	Standardized mean diff.	Standardized mean diff., weighted
**Age in years**,** median (IQR)**	49 (36–60)	49 (36–60)	-	-
**Age class**				
≤ 40 years	39,806 (32.1)	39,841 (32.1)	-	-
41–60 years	53,823 (43.4)	53,889 (43.4)	-	-
> 60 years	30,397 (24.5)	30,420 (24.5)	-	-
**Sex**				
Female	66,824 (53.9)	66,783 (53.8)	-	-
Male	57,202 (46.1)	57,367 (46.2)	-	-
**Deprivation Index**,** mean (SD)**	3.0 (0.6)	3.1 (0.6)	0.07588	0.00002
**Chronic comorbidities**				
Chronic kidney disease	1502 (1.2)	1158 (0.9)	0.02703	0.00043
Chronic kidney disease - Dialysis	645 (0.5)	262(0.2)	0.05122	-0.00084
Tumor follow-up	8585 (6.9)	7236 (5.8)	0.04477	-0.00029
Tumor in treatment	6504 (5.2)	4617 (3.7)	0.07377	-0.00006
Hypertension	26,847 (21.6)	26,725 (21.5)	0.00291	0.00000
Ischemic Heart Disease	4148 (3.3)	3562 (2.9)	0.02740	-0.00025
Valvular Heart Disease	1447 (1.2)	1231 (1.0)	0.01695	0.00000
Arrhythmic cardiomyopathy	5439 (4.4)	4757 (3.8)	0.02790	-0.00008
Non-Arrhythmic cardiomyopathy	3788 (3.1)	3402 (2.7)	0.01872	-0.00007
Congestive heart failure	3861 (3.1)	2953 (2.4)	0.04496	-0.00045
Peripheral artery disease	1395 (1.1)	997 (0.8)	0.03293	-0.00017
Venous disorders	604 (0.5)	525 (0.4)	0.00953	-0.00020
Cerebrovascular disease	3051 (2.5)	2547 (2.1)	0.02751	0.00015
Asthma	4446 (3.6)	4823 (3.9)	-0.01583	0.00000
COPD	5597 (4.5)	5591 (4.5)	0.00045	0.00000
Chronic respiratory failure	332 (0.3)	261 (0.2)	0.01177	0.00000
Obesity	1576 (1.3)	1828 (1.5)	-0.01734	0.00000
**Influenza vaccination**				
2017	12,982 (10.5)	12,753 (10.3)	0.00639	0.00013
2018	15,301 (12.3)	14,738 (11.9)	0.01428	0.00004
2019	17,655 (14.2)	16,780 (13.5)	0.02080	-0.00007
**Pneumococcal vaccination**	3827 (3.1)	3326 (2.7)	0.02431	-0.00009

Abbreviations: IQR, interquartile range; COPD, chronic obstructive pulmonary disease; SD, standard deviation

Differences in the association between exposure to SARS-CoV-2 and diabetes across sexes and age classes were first described by weighted Kaplan-Meier survival curves and then evaluated by stratified analysis with a weighted Cox model. Age was divided into three groups, namely, ≤ 40, 41–60, and > 60 years, for comparability with other studies.

The hazard ratio (HR) was provided as a risk measure with a 95% confidence interval (CI). The proportional hazards assumption was evaluated graphically and through Schoenfeld residuals analysis.

All the analyses were carried out using SAS/BASE and SAS/STAT software, version 9.4 (PROC PSMATCH for the IPW logistic model; PROC LIFETEST and PROC PHREG for survival analysis).

## Results

The number of residents in the ATS area and tested for SARS-CoV-2 infection who met the inclusion criteria was 590,931, and the total number of tests performed in the selected period was 1,175,761 (Fig. 1). After excluding tests if the individual developed diabetes within 30 days and considering the first positive test and negative test not preceded by a positive test, we found 127,024 individuals with a positive test (exposed) and 795,975 negative tests for 490,340 individuals (not exposed). After matching, 125,587 (98.9% of 127,024) positive individuals were paired with negative individuals. One hundred thirty pairs were excluded because the negative individual became positive before the positive individual was enrolled within the same week. An additional 2738 individuals (1431 negative and 1307 positive) were excluded because of missing data on the deprivation index.

The final study cohort included 248,176 participants: 124,026 SARS-CoV-2-negative individuals and 124,150 SARS-CoV-2-positive individuals (97.7% of 127,024). Approximately 54% were female (53.9% among negative individuals and 53.8% among positive individuals). The mean age was 49.6 years (± 18.3) for both the positive and negative groups. The baseline characteristics of the study cohort are reported in Table 1. A total of 55.2% and 24.0% of the positive individuals were fully and partially vaccinated, respectively, during follow-up. Among negative individuals, the percentages were 77.2% (fully vaccinated) and 3.4% (partially vaccinated). The percentage of unvaccinated individuals was very similar between positive and negative groups (20.9% vs. 19.4%, respectively). The median time from enrollment to the first dose was slightly longer for positive individuals (209 days; IQR 168–249) than for negative individuals (196 days; IQR 137–238).

The median follow-up was 415 days (IQR 385–430), with a maximum value of 670 days for both negative and positive groups. There were 18,162 (14.6%) negative individuals who became positive during follow-up, but only 7891 (6.3% of 125,587) became positive before 31 December 2020 and were matched with a negative individual and included in the study as positive. This number was further reduced to 7170 after the exclusions described above (5.8% of 124,150). Deaths were 2.5% among those who tested positive, and 1.9% among those who tested negative. The percentage of negative individuals whose follow-up ended because of death varied from 0.93% for fully vaccinated individuals to 2.93% for partially vaccinated individuals and 8.41% for unvaccinated individuals. The percentages among positive individuals were 0.78%, 0.48% and 6.42%, respectively. The individuals lost to follow-up were 3172, which is 1.3% of the entire sample, with equal percentages among positive and negative individuals.

During the follow-up period, 739 (0.60%) positive and 657 (0.53%) negative individuals developed diabetes. The median time to diabetes detection was 171 days (IQR 82–322) in the positive group and 190 days (IQR 105–310) in the negative group. The incidence among positive individuals was 572.82 per 100,000 person-years (CI 531.52–614.12), and that among negative individuals was 509.50 per 100,000 person-years (CI 470.54–548.46). The distribution of diabetes diagnoses varied differently with respect to vaccination status between test-negative and test-positive groups (Table 2): 0.26% vs. 0.21% among fully vaccinated individuals; 1.30% vs. 0.50% among partially vaccinated individuals; and 1.49% vs. 1.74% among unvaccinated individuals.

**Table 2:** Follow-up characteristics of the two study groups stratified by vaccination status

*n* (%)	SARS-CoV-2 negative (*n* = 124026)			SARS-CoV-2 positive (*n* = 124150)		
	2 doses	1 dose	Not vaccinated	2 doses	1 dose	Not vaccinated
Censored*	88,357 (92.24)	3218 (77.19)	9011 (37.44)	65,572 (95.76)	28,459 (95.64)	21,390 (82.52)
Dead	894 (0.93)	122 (2.93)	2023 (8.41)	533 (0.78)	143 (0.48)	1664 (6.42)
Diabetes	245 (0.26)	54 (1.30)	358 (1.49)	141 (0.21)	148 (0.50)	450 (1.74)
Infected/Reinfected	5619 (5.87)	633 (15.18)	11,910 (49.48)	1700 (2.48)	708 (2.38)	1652 (6.37)
Lost to follow-up	673 (0.70)	142 (3.41)	767 (3.19)	527 (0.77)	299 (1.00)	764 (2.95)

* Individuals observed until 31 December 2021 or until death, detection of diabetes, infection/reinfection of the matched individual

The values are the numbers (percentages)

The weights for the Cox model, calculated through IPW, varied between 0.608 and 2.828, with a mean value close to 1 for both positive and negative groups. Therefore, the difference in the mean weight between the positive and negative groups was nearly zero. The variables used to evaluate the weights were well balanced between the positive and negative groups after weighting. The standardized mean differences of the weighted variables were close to zero (Table 1; Supplementary Figure S2), and the variance ratios were close to 1 and in the interval (0.5–2) for all the variables.

**Fig. 2:**
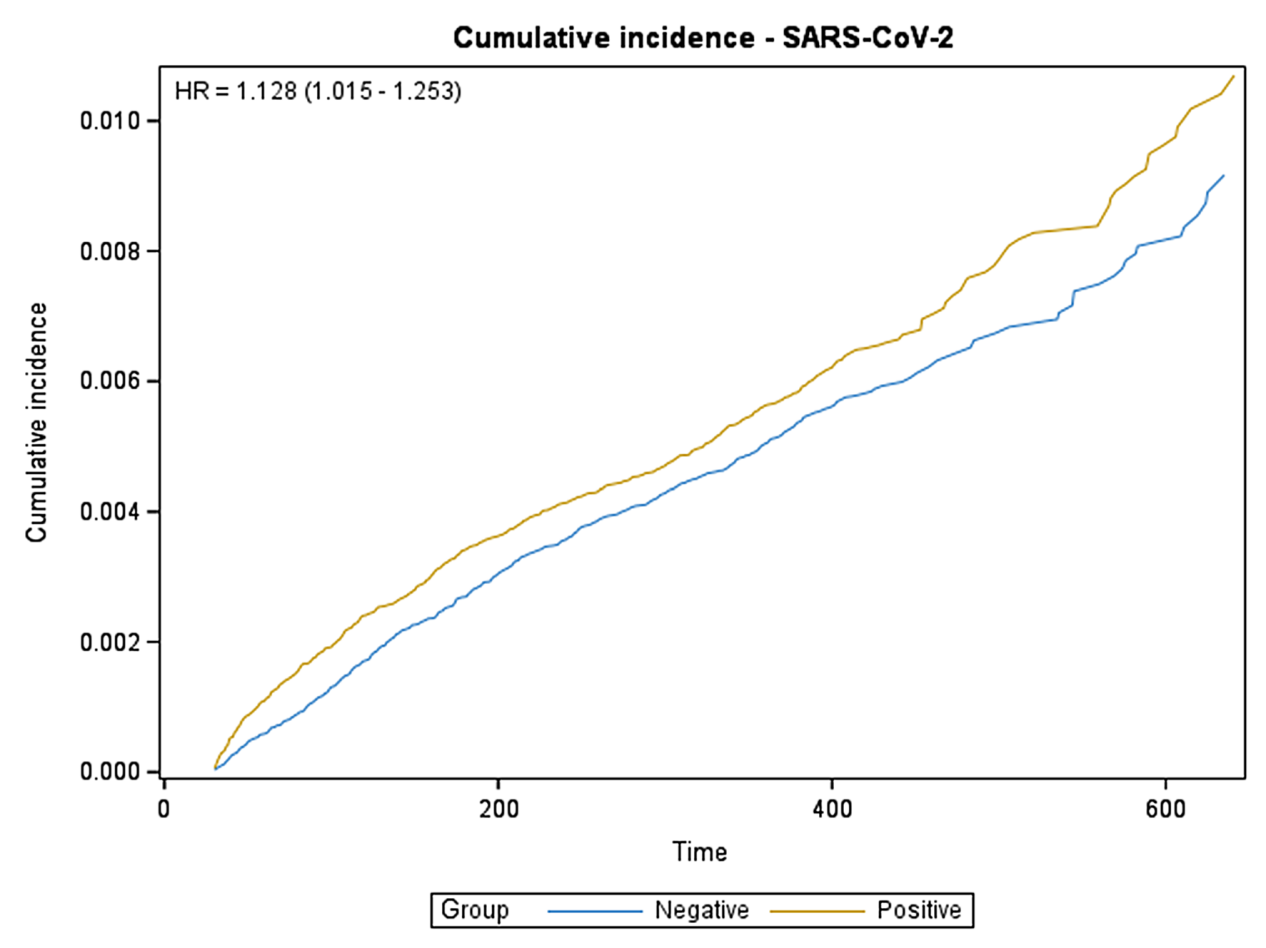
Cumulative incidence of diabetes in positive and negative individuals

**Fig. 3:**
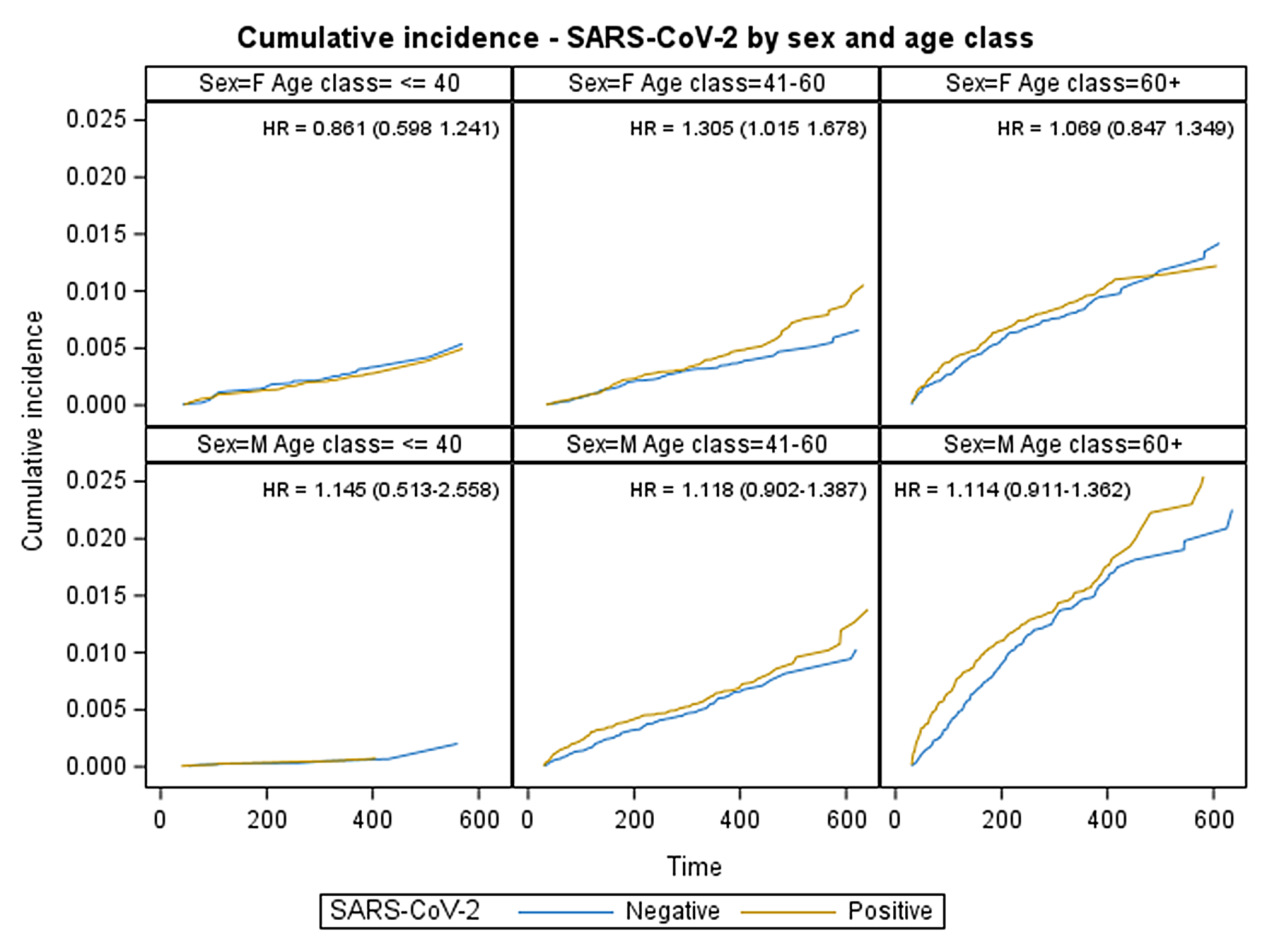
Cumulative incidence of diabetes in positive and negative individuals stratified by age and sex

The overall hazard ratio (HR) for diabetes was 1.128 (CI 1.015–1.253) for individuals in the positive group compared with the negative group; the cumulative incidence of the two groups is represented in Fig. 2 (see also panel A in Supplementary Figure S3 for the log–log survival curve). Sex-stratified analyses yielded effect estimates of similar magnitude for both males (HR 1.138; CI 0.985–1.312) and females (HR 1.115; CI 0.956–1.301), with confidence intervals centred on values above 1, although non-significant. The effects of SARS-CoV-2 infection within different age and sex strata are shown in Fig. 3 (see also panel B in Supplementary Figure S3 for the log–log survival curves). While estimates of HRs were comparable across age classes in males, results in females were more heterogeneous, with the subgroup aged 41–60 years showing the highest risk estimate that reached statistical significance (HR 1.305; CI 1.015–1.678).

## Discussion

In this population-based study in Italy during the first pandemic wave, we observed an increased risk of detecting long-term diabetes following SARS-CoV-2 infection in the overall adult population. The effect of infection on incident diabetes varied by sex and age group, with the strongest association observed among women aged 41–60 years.

The interplay between diabetes and COVID-19 exacerbates health outcomes through biological and social interactions and increases their burden on vulnerable populations, constituting a syndemic [19]. However, the reasons for the relationship between COVID-19 and newly detected diabetes have yet to be clarified [13]. Potential explanations for the diabetogenic effect of COVID-19 include impaired insulin secretion as a direct consequence of viral tropism for pancreatic beta cells, autonomic dysfunction, a hyperactivated immune response and a persistent proinflammatory state leading to insulin resistance, as well as the hyperglycemic effects of corticosteroids used to treat infection [13, 14, 20]. While these factors are likely to act as acute stressors that trigger overt manifestations of diabetes in predisposed individuals, it is also likely that the clinical, social and behavioral risk factors for the two conditions overlap. Specific considerations should be made for different diabetes subtypes, especially type 1 and type 2. The results reported in the literature regarding the associations between infection and different subtypes are quite contrasting [12]. Consistent with other studies [12, 13], we defined diabetes mellitus as our outcome and did not differentiate between diabetes subtypes. While case-detection of overall diabetes using population-level HADs is accurate, algorithms for the classification of specific subtypes are less reliable. As our cohort was drawn from an adult population, we expect the bulk of this signal to be type 2 diabetes, which is consistent with findings such as those reported by Xie et al. [13].

Our overall results were consistent with other study findings, which present an increased risk of incident diabetes in infected individuals compared with noninfected individuals, although with different values of risk [2, 5, 12, 13].

Naveed et al. [12] conducted a study of the adult population of British Columbia, Canada, in the years 2020–2021. With an overall median follow-up time of 257 days and a median age of 32 years (IQR 25–42), the authors estimated an HR of 1.17 (1.06–1.28). The difference in median age could be explained by the authors’ decision to exclude residents of long-term care facilities. Moreover, we adopted a study design that involved discontinuing follow-up of the entire pair when the negative individual became positive. It seems that Naveed et al. [12] did not clearly specify whether follow-up of positive individuals continued when negative individuals in the pair became positive and whether the latter were reintroduced into the analysis as positive. This difference in design might explain the lower HR value found in our study. Naveed et al. [12] also evaluated the incidence among negative and positive groups. The incidence value for unexposed individuals (negative) was 508.7 per 100,000 person-years (CI 485.6–531.8), which is comparable to the value we calculated for our cohort (509.5 per 100000 person-years; CI 470.5–548.5). Instead, the incidence calculated in the positive group (672.2 per 100000 person-years; CI 618.7–725.6) was higher than our estimate (572.8 per 100000 person-years; CI 531.5–614.1), which is consistent with their higher reported HR.

In their study, Xie et al. [13] analyzed incident diabetes using the national databases of the US Department of Veterans Affairs. With a median follow-up of 352 (245–406) days, they estimated an HR equal to 1.40 (1.36–1.44) for exposed individuals, identified by a positive test, against a contemporary control group, identified as non-positive individuals. Given the nature of the population under study, the proportion of males was greater than 88%, and the mean age was approximately 61 years for both the exposed and unexposed groups. These differences in demographic characteristics could at least in part explain the differences with our findings.

Mizrahi et al. [5] conducted a nationwide cohort study using electronic medical records from a healthcare organization with a study design similar to ours. However, they excluded patients admitted to the hospital within 30 days of infection to evaluate outcomes after mild SARS-CoV-2 infection and focused on unvaccinated individuals. Moreover, the enrollment period included wild-type and alpha variants of SARS-CoV-2. They reported an HR of 1.28 (1.05–1.57) for the follow-up period of 180–360 days in 140,991 individuals and a nonsignificant HR for the period of 30–180 days. The restriction of the follow-up period, the inclusion of different virus variants, and possible differences in population characteristics could explain the higher HR estimate. Moreover, we can observe that the confidence interval calculated by the authors largely overlaps with ours.

Daugherty et al. [2] investigated a wide range of clinical sequelae, including type 2 diabetes, in a population aged 18–65 years via commercial insurance databases. Exposed individuals, defined on the basis of a positive test or COVID-19 diagnosis or hospital admission, were compared with all individuals without these criteria. The comparison was performed at the 6-month follow-up (95 days of median follow-up for the positive group), and the estimated HR was 1.83 (1.60–2.10). This value was the highest reported in the literature of population-based studies. We suggest that the differences in our findings can be explained by the specificity of the study design and target population.

In both studies, Naveed et al. [12] and Mizrahi et al. [5] reported a significant effect of infection in males (HR 1.22, CI 1.06–1.40, and HR 1.33, CI 1.06–1.68, respectively) whereas estimates for females were closer to the null. In our analysis, the HR in males was of comparable magnitude although the confidence interval included unity (1.138; CI 0.985–1.315). This result could be due to a lower number of included individuals because of a shorter enrollment period or 1:1 matching. The latter choice was made because the number of negative tests was small compared with the number of positive tests during periods of low viral circulation (Supplementary Figure S1).

We could not find population-based studies that performed stratified analyses for both sex and age classes, specifically for the association between SARS-CoV-2 and diabetes. Our findings suggest a stronger association between infection and diabetes among females aged 41–60 years (HR 1.305; CI 1.015–1.678). In the other strata, aged over 40, a tendency towards a positive association can be observed. In the age-class stratified analysis, Mizrahi et al. [5] reported a significant effect of infection in people aged over 60 (HR 1.82; CI 1.13–2.95) only. However, in their analysis of several symptoms and clinical conditions, they reported that those aged 41–60 years had the highest number of long-term COVID-19 health outcomes that were significantly elevated during the year after infection. Even though diabetes was not significantly related to infection, in their study, those aged 41–60 years were more prone to presenting infection-related symptoms, suggesting a peculiar susceptibility of this subgroup. Moreover, Estiri et al. [3] reported an association between COVID-19 and diabetes in both 3–6-month and 6–9-month periods and in the 3–6-month period for people aged < 65 years.

The strengths of our study include the enrollment of a large number of individuals from the general population without particular selection criteria and the rigorous study design. Using routinely collected data, we were able to consider both the clinical and sociodemographic conditions of a large population to adjust the evaluation of risk. Moreover, adjustment via IPW is performed without any use of the outcome, preventing model specification from being influenced by adjustment effects on the outcome. While selection bias may arise from restrictions inherent to the matching process, the high uptake of testing in Lombardy during the study period (across age classes, clinical conditions, and settings) and the very high matching coverage (98.9%) suggest that no specific population subgroups were systematically excluded.

Although the main conditions potentially associated with infection and the onset of diabetes have been considered, we cannot exclude that a residual amount of confounding potentially related to lifestyle factors or physiological/anthropometric parameters may have remained uncontrolled, which is a potential limit of the study. Differences by race or ethnicity in both COVID-19 outcomes and diabetes risk are well documented [13, 21]. However, this information was not available in our demographic database and could not be included among the covariates in the IPW model. Furthermore, we cannot rule out that the observed effect is at least partly due to detection bias, in the form of an anticipation of diagnosis or opportunistic diagnosis in individuals who underwent tight clinical surveillance to monitor sequelae of COVID-19. The use of HAD-based case-detection algorithm implies that the tracked incidence reflects identification of diabetes generated by a new contact with the health system fulfilling specific characteristics (hospitalization for diabetes, prescription of a drug or activation of an exemption which must be consequent to a physician visit), rather than biological onset. Any differential healthcare utilization between groups, especially in the early stages of the pandemic, may influence the magnitude of the observed HR. In fact, diabetes detection may differ according to healthcare-seeking behavior or closer follow-up by clinicians of individuals with SARS-CoV-2 infection. This source of differential outcome misclassification could lead to an overestimation of the association between infection and incident diabetes. However, the exclusion of individuals who were detected with diabetes within 30 days from the test (indicating an immediate opportunistic diagnosis) and the modest difference in time-to-diagnosis between test-positive and test-negative individuals (170 vs. 190 days) would suggest that differential detection is unlikely to fully explain the observed hazard ratio over the entire follow-up. Although 75% of participants had more than one year of follow-up, it cannot be excluded that delays in HAD-based diabetes detection partially affect the observed differences between groups. However, the effect of this possible delay cannot be quantified within this study design. While HAD-based case-detection algorithms for diabetes mellitus are quite accurate, the detection of prediabetic conditions remains a challenge. In general, we cannot rule out the possibility of undiagnosed prediabetic or diabetic conditions in both positive and negative groups. Finally, despite the introduction of restrictive measures for accessing public places, a limitation in common with other studies is the impossibility of identifying infected individuals who have not been tested, for example, because they were completely asymptomatic.

Because of the study design, individuals were always subjected to COVID-19 vaccination between enrollment, which was the date of testing, and the observed events (i.e., diabetes detection). Thus, it was not possible to observe the effect of vaccination on SARS-CoV-2 infection and the possible modifications induced in the relationship between SARS-CoV-2 infection and diabetes. Vaccination was not considered in the IPW adjustment, where only baseline characteristics were considered. Although a direct effect of COVID-19 vaccination on diabetes onset has not been documented in large-scale studies [22], we cannot rule out some indirect association. The different distributions of deaths between the positive and negative groups are consistent with the ability of vaccination to protect against adverse consequences of infection. The different distributions of diabetes diagnoses, which decreases with different degrees of vaccination coverage between positive and negative groups, suggest the presence of individuals who were infected but were not tested. The possibility that individuals escaped test detection and were not correctly classified as positive represents a limitation of this study, which is shared by other studies of the same nature. The quantification of this phenomenon needs to be evaluated through specific study designs and analyses.

## Conclusions

This study was conducted on a large general population in one of the territories most and earliest affected by the pandemic in Italy and at the international level. Given the limited number of studies conducted on the general population and their discordant results, this work aims to contribute to generating evidence about the association between SARS-CoV-2 infection and newly detected diabetes, overcoming some limitations in the literature with a rigorous study design. Our results suggest an increased risk for SARS-CoV-2-positive individuals in the general population compared with SARS-CoV-2-negative individuals, with a stronger association in women aged 41–60 years. Mechanistic pathways, including inflammation, corticosteroid use, and unmasking of pre-existing prediabetes, may underlie these associations. These findings underscore the importance of ongoing surveillance of metabolic health in populations recovering from COVID-19, as the growing burden posed by diabetes on healthcare systems calls for targeted monitoring and preventive strategies in populations at risk.

## Electronic Supplementary Material

Below is the link to the electronic supplementary material.

Supplementary Material 1: Figure S1 – Number of tests in the enrollment period.

Supplementary Material 2: Figure S2 – Standardized mean difference before and after weighting.

Supplementary Material 3: Figure S3 – Log–log survival curves.

Supplementary Material 4: Table S1 – Criteria for case-detection of diabetes from routinely collected health data.

## Data Availability

The individual data analyzed in the current study contain sensitive personal information; therefore, they are not publicly available due to privacy issues according to the European Regulation (EU) 2016/679 and Italian Legislative Decree no. 101/2018. Aggregate datasets underlying this article will be shared upon reasonable request to the corresponding author or to epidemiologia@ats-milano.it.

## Declarations

### Patient and public involvement

Owing to the nature of this study and data privacy constraints, patients and members of the public were not involved in the design, analysis, or interpretation of the study.

### PASCNET (Post-Acute Sars-Cov-2 syndrome NETwork) study group composition

Claudio Lucifora^3^, Daria Vigani^3,4^, Federico Franzoni^3^, Gabriele Letta^3^, Laura Antolini^2^, Giuseppe Lapadula^2^, Elena Tassistro^2^, Maria Grazia Valsecchi^2^, Stefano Denicolai^4^, Marica Grego^4^, Diala Kabbara^4^, Costanza Baldrighi^4^, Antonio Giampiero Russo^1^, Pietro Magnoni^1^, Cristina Mazzali^1^, Alberto Milanese^1^, Rossella Murtas^1^, Andrea Salvatori^1^, Deborah Testa^1^, Sara Tunesi^1^, Adele Zanfino^1^, Simona Dalle Carbonare^5^, Federica Manzoni^5^, Simona Migliazza^5^, Pietro Giovanni Perotti^5^, Linda Guarda^6^, Marco Villa^6^, Silvia Tillati^7^, Giacomo Crotti^7^, Giuseppe Sampietro^7^, Alberto Zucchi^7^, Anita Andreano^8^, Luca Cavalieri d’Oro^8^, Elisabetta Merlo^8^, Magda Rognoni^8^, Piersimone Fontana^9^, Giovanni Maifredi^9^, Ivan Cometti^10^, Anna Clara Fanetti^10^, Maria Letizia Gambino^11^, Monica Lanzoni^11^, Giuseppe Emanuele La Piana^12^, Anna Bussi^13^, Vincenzo Belcastro^14^, Stefano Rusconi^15^, Luigi Magnani^16^, Maurizio Morlotti^17^, Andrea Patroni^17^, Raffaele Bruno^18^, Elisabetta Pagani^18^, Paolo Sacchi^18^ and Valentina Zuccaro^18^.

^1^Epidemiology Unit, Agency for Health Protection Milan, Milan, Italy.

^2^University of Milano-Bicocca, School of Medicine and Surgery and Bicocca Bioinformatics Biostatistics and Bioimaging Centre (B4), Milan, Italy.

^3^Department of Economics and Finance, Catholic University of the Sacred Heart, Milan, Italy.

^4^Department of Economics and Management, University of Pavia, Pavia, Italy.

^5^Epidemiology Unit, Agency for Health Protection Pavia, Pavia, Italy.

^6^Epidemiology Unit, Agency for Health Protection Val Padana, Cremona, Italy.

^7^Epidemiology Unit, Agency for Health Protection Bergamo, Bergamo, Italy.

^8^Epidemiology Unit, Agency for Health Protection Brianza, Monza, Italy.

^9^Epidemiology Unit, Agency for Health Protection Brescia, Brescia, Italy.

^10^Epidemiology Unit, Agency for Health Protection Montagna, Sondrio, Italy.

^11^Epidemiology Unit, Agency for Health Protection Insubria, Varese, Italy.

^12^Respiratory Rehabilitation Unit, ASST Crema – Ospedale Santa Marta Rivolta D’Adda, Crema, Italy.

^13^General Medicine Unit, ASST del Garda – Presidio Ospedaliero di Manerbio/Leno.

^14^Neurology Unit, ASST Lodi – Ospedale Maggiore di Lodi, Lodi, Italy.

^15^Infectious Diseases Unit, ASST Ovest Milanese – Ospedale di Legnano, Legnano, Italy.

^16^Internal Medicine Unit, ASST Pavia – Ospedale Civile di Voghera, Voghera, Italy.

^17^ASST Valcamonica, Brescia, Italy.

^18^Fondazione IRCCS Policlinico San Matteo, Pavia, Italy.

## Abbreviations

ATS: *Agenzia di Tutela della Salute* – Agency for Health Protection
CI: 95% confidence interval
HAD: Health administrative database
IPW: Inverse probability weighting
IQR: Interquartile range
HR: Hazard ratio
