# Supplementary material for: Incident diabetes within the first two years after SARS-CoV-2 infection: a population-based retrospective cohort study of the Agency for Health Protection of Milan, Italy
Source: BMC Infect Dis. 2026 May 11;26:1240. doi: 10.1186/s12879-026-13467-4 (PMC13335262; doi:10.1186/s12879-026-13467-4)
Supplement: Supplementary file 1 — Supplementary Material 1: Figure S1 – Number of tests in the enrollment period. [file 12879_2026_13467_MOESM1_ESM.pdf]

Incident diabetes within the first two years after SARS-CoV-2 infection: a population-based retrospective cohort study of the Agency for Health Protection of Milan, Italy

Supporting information: Supplementary Figure S1

Tests performed daily between 1/3/2020 and 31/12/2020 and ratio of number of positive and negative tests.

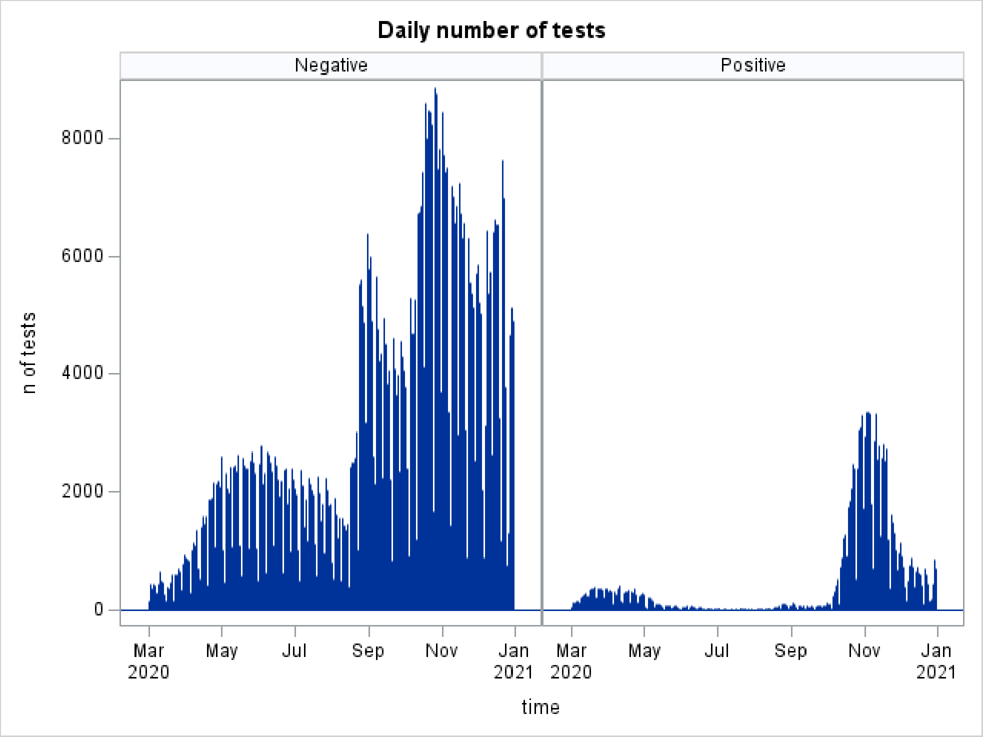

A) Daily number of tests starting from March, 2020 to December, 2020 divided by test result.

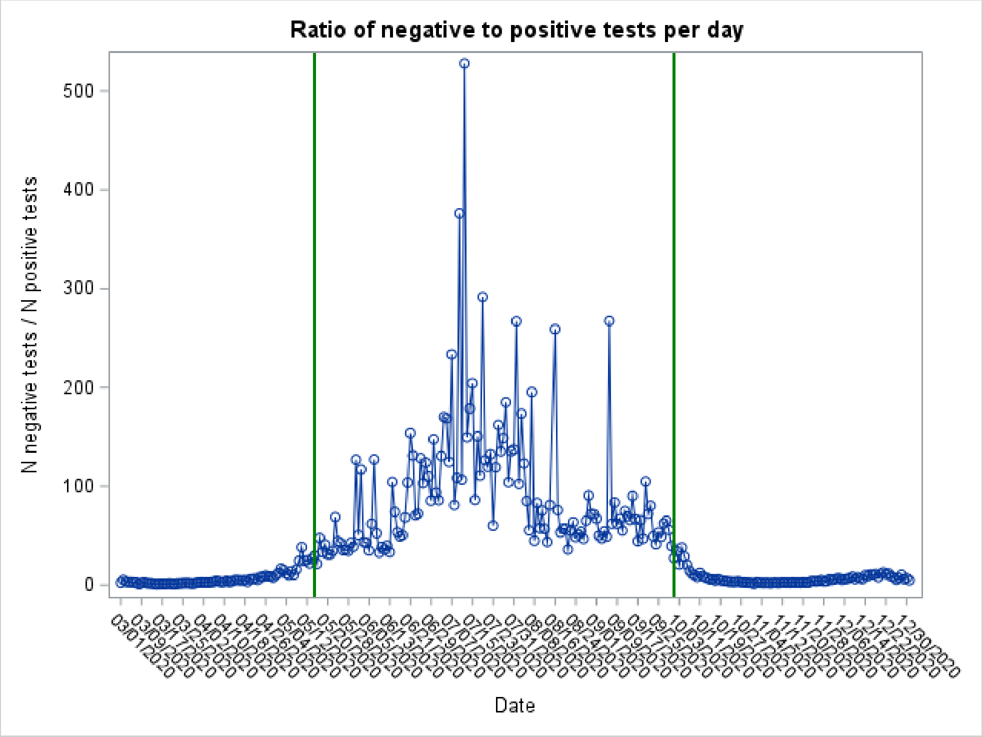

B) Ratio of negative vs positive test number on daily basis from March 1, 2020 to December 31, 2020.

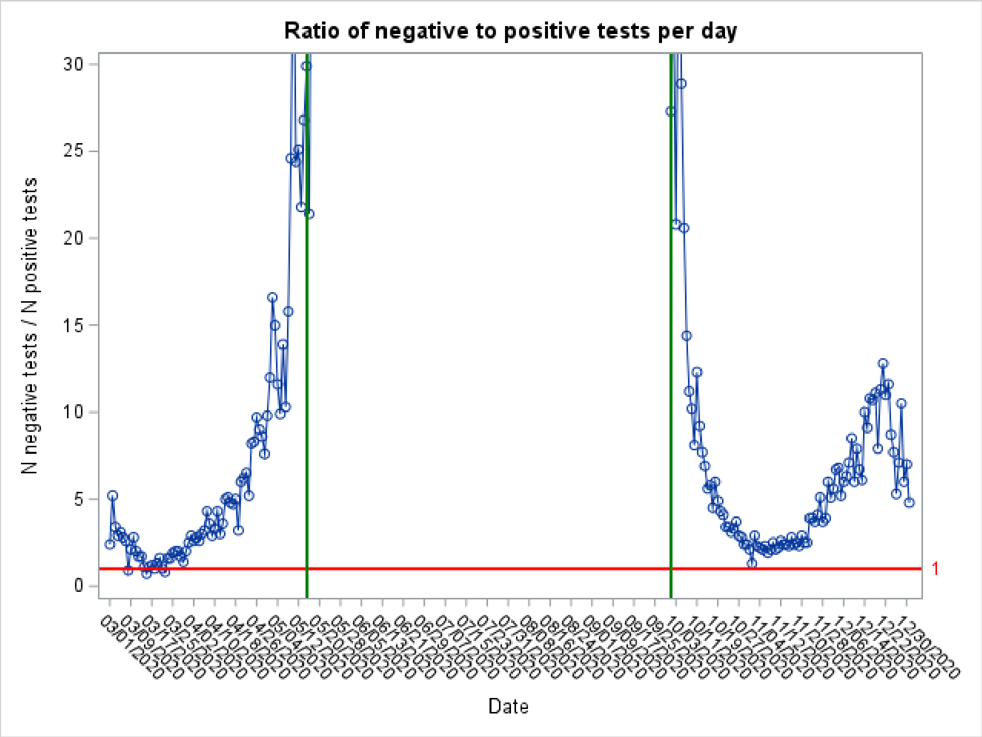

C) Ratio of negative vs positive test number on daily basis focusing on periods from March 1, 2020 to May 16, 2020 and from October 1, 2020 to December 31, 2020. The reference red line is set to ratio = 1.
