# Supplementary material for: Incident diabetes within the first two years after SARS-CoV-2 infection: a population-based retrospective cohort study of the Agency for Health Protection of Milan, Italy
Source: BMC Infect Dis. 2026 May 11;26:1240. doi: 10.1186/s12879-026-13467-4 (PMC13335262; doi:10.1186/s12879-026-13467-4)
Supplement: Supplementary file 2 — Supplementary Material 2: Figure S2 – Standardized mean difference before and after weighting. [file 12879_2026_13467_MOESM2_ESM.pdf]

Incident diabetes within the first two years after SARS-CoV-2 infection: a population-based retrospective cohort study of the Agency for Health Protection of Milan, Italy

Supporting information: Supplementary Figure S2

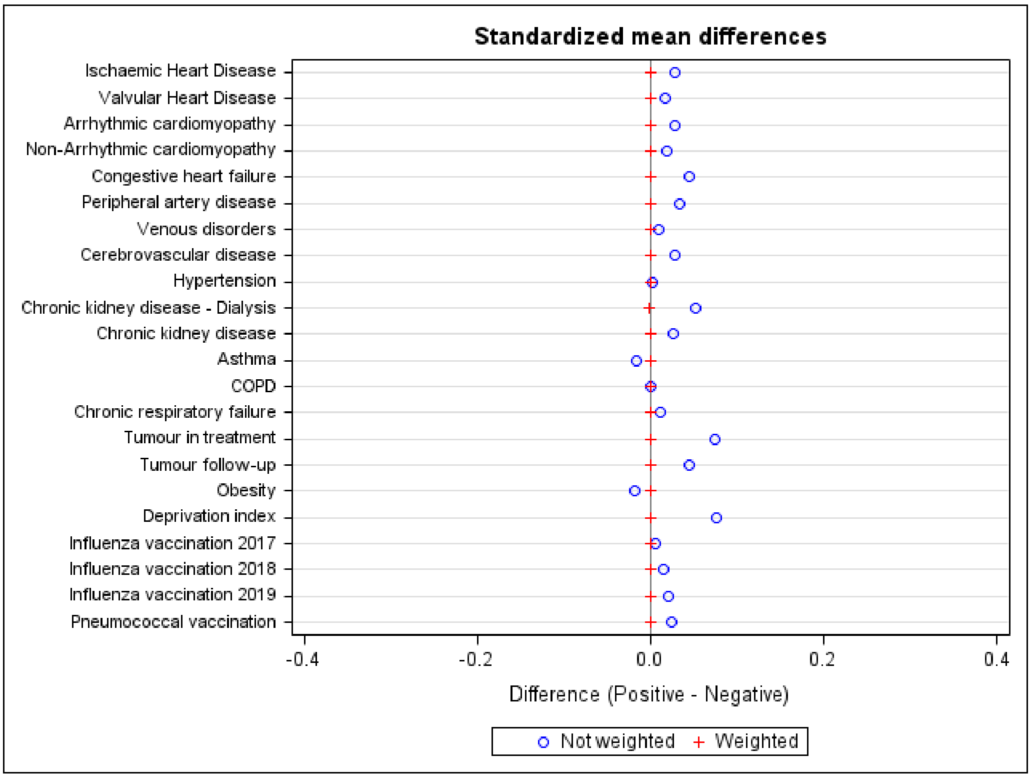

Standardized mean differences of covariates before and after weighting using IPW.
