# Supplementary material for: Incident diabetes within the first two years after SARS-CoV-2 infection: a population-based retrospective cohort study of the Agency for Health Protection of Milan, Italy
Source: BMC Infect Dis. 2026 May 11;26:1240. doi: 10.1186/s12879-026-13467-4 (PMC13335262; doi:10.1186/s12879-026-13467-4)
Supplement: Supplementary file 3 — Supplementary Material 3: Figure S3 – Log–log survival curves. [file 12879_2026_13467_MOESM3_ESM.pdf]

Incident diabetes within the first two years after SARS-CoV-2 infection: a population-based retrospective cohort study of the Agency for Health Protection of Milan, Italy

Supporting information: Supplementary Figure S3

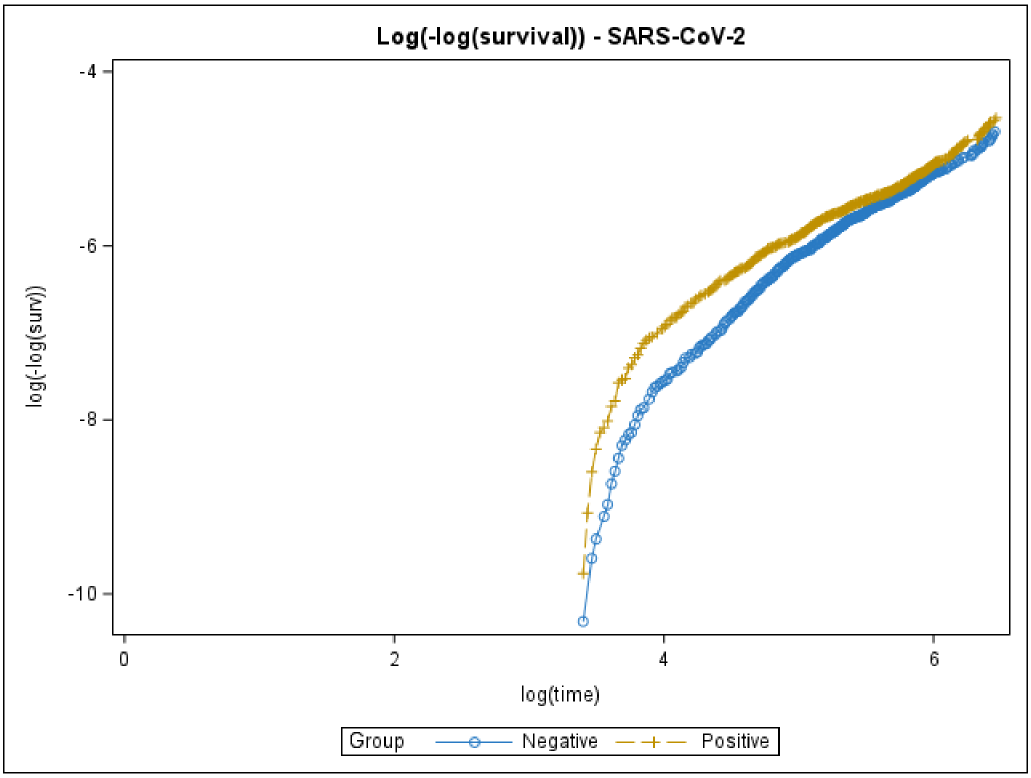

A) Log(-log(survival) curves (weighted) by test result.

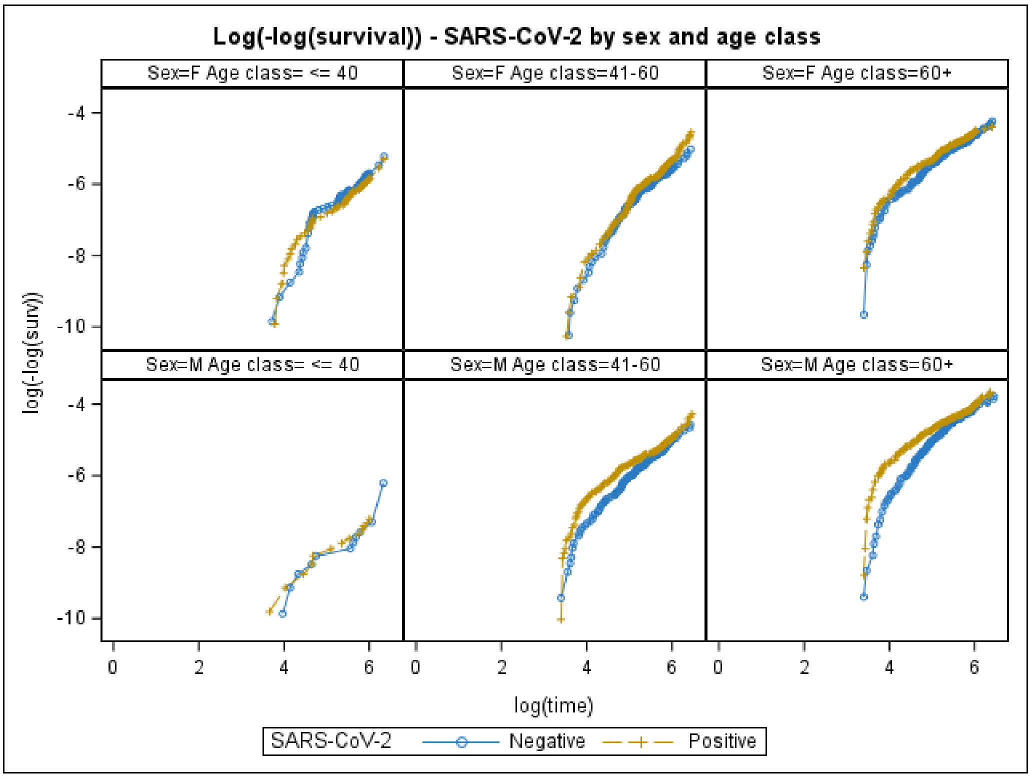

B) Log(-log(survival) curves (weighted) by sex, age class and test result.
