# Supplementary material for: Incident diabetes within the first two years after SARS-CoV-2 infection: a population-based retrospective cohort study of the Agency for Health Protection of Milan, Italy
Source: BMC Infect Dis. 2026 May 11;26:1240. doi: 10.1186/s12879-026-13467-4 (PMC13335262; doi:10.1186/s12879-026-13467-4)
Supplement: Supplementary file 4 — Supplementary Material 4: Table S1 – Criteria for case-detection of diabetes from routinely collected health data. [file 12879_2026_13467_MOESM4_ESM.pdf]

**Incident diabetes within the first two years after SARS-CoV-2 infection: a population-based retrospective cohort study of the Agency for Health Protection of Milan, Italy**

| Database | Coding system and thresholds | Code(s) | Lookback period (years) |
|---|---|---|---|
| HDR | DRG | 294 (diabetes, age > 35 years);<br>295 (diabetes, age < 36 years) | 0-5 |
| | ICD-9-CM Diagnosis (any field) | 250.0x (uncomplicated DM);<br>250.1x (DM with ketoacidosis);<br>250.2x (DM with hyperosmolar hyperglycemic state);<br>250.3x (DM with other type of coma);<br>250.4x (DM with renal complications);<br>250.5x (DM with eye complications);<br>250.6x (DM with neurological complications);<br>250.7x (DM with peripheral vascular complications);<br>250.8x (DM with other specified complications);<br>250.9x (DM with unspecified complications);<br>357.2 (diabetic polyneuropathy);<br>362.01-362.02 (diabetic retinopathy) | 0-5 |
| Exemption database | National exemption coding system | 013.250 | 0-10 |
| Drug dispensing databases | ATC (>=50% of DDDs covered) | A10A* (insulin);<br>A10B* (oral antidiabetic agents) | 0-1 |

**Supplementary Table S1.** Extrapolation of the list of criteria in use in Lombardy Region (Deliberation of Regional Council no. 6164/2017 and subsequent amendments and supplements) to build the Chronic Disease Database from routinely collected health data. Subjects fulfilling ≥ 1 of the following criteria are classified as affected by diabetes mellitus.

Abbreviations: ATC, World Health Organization Anatomical Therapeutic Chemical classification system; DDD, defined daily doses; DM, diabetes mellitus; DRG, diagnosis-related group; HDR, hospital discharge records; ICD-9-CM, International Classification of Diseases, 9th Revision, Clinical Modification.
